# A preliminary taxonomy and a standard knowledge base for mental-health system indicators in Spain

**DOI:** 10.1186/1752-4458-4-29

**Published:** 2010-12-01

**Authors:** Luis Salvador-Carulla, José Alberto Salinas-Pérez, Manuel Martín, Mont-serrat Grané, Karina Gibert, Miquel Roca, Antonio Bulbena

**Affiliations:** 1 Asociación Científica PSICOST, Jerez de la Frontera, Spain; 2 Psiquiatría, Departamento de Neurociencias, Universidad de Cádiz, Cádiz, Spain; 3 ETEA-Facultad de Económicas y Empresariales, Córdoba, Spain; 4 Instituto de Investigaciones Psiquiátricas, Fundación Mª Josefa Recio, Bilbao, Spain; 5 Institut de Neuropsiquiatria i Addiccions, Parc de Salut MAR, Barcelona, Spain; 6 Dep. Estadística e Investigación Operativa, Universitat Politècnica de Catalunya, Barcelona, Spain; 7 Institut Universitari d'Investigació en Ciencies de la Salut (IUNICS), Universitat de les Illes Balears, Palma de Mallorca, Red de Investigación en Actividades Preventivas y de Promoción de la salud (RedIAPP; 8 Sociedad Española de Psiquiatría (Spanish Society of Psychiatry), Clinical Management Working Group (GClin-SEP) with the collaboration of PSICOST research association and RedIAPP

## Abstract

**Background:**

There are many sources of information for mental health indicators but we lack a comprehensive classification and hierarchy to improve their use in mental health planning. This study aims at developing a preliminary taxonomy and its related knowledge base of mental health indicators usable in Spain.

**Methods:**

A qualitative method with two experts panels was used to develop a framing document, a preliminary taxonomy with a conceptual map of health indicators, and a knowledge base consisting of key documents, glossary and database of indicators with an evaluation of their relevance for Spain.

**Results:**

A total of 661 indicators were identified and organised hierarchically in 4 domains (Context, Resources, Use and Results), 12 subdomains and 56 types. Among these the expert panels identified 200 indicators of relevance for the Spanish system.

**Conclusions:**

The classification and hierarchical ordering of the mental health indicators, the evaluation according to their level of relevance and their incorporation into a knowledge base are crucial for the development of a basic list of indicators for use in mental health planning.

## Background

Health indicators are tools designed to measure the health status of people and the functioning of health services through the various factors that influence them (demographic, economic, social) [1,2]. These provide the basic information for system analysis and decision-making in policies, planning and health management. The area of mental health presents added difficulties for the development of a useful list of health indicators for a variety of reasons. Firstly, this is a complex area in which health, social, educational and criminal and justice services coexist, where the care teams are multidisciplinary, and in which an integral care focus should be adopted [3]. Secondly, there are no reliable biological indicators for either the disorders assessed or the results, which complicates epidemiological and outcome research. Thirdly, mental health has been included late into the general health system (in Spain from 1986), it presents problems of under-financing and the lack of national data bases which exists in other disciplines (e.g. Oncology or AIDS) [4].

The instruments which compile indicators are rarely organised as a knowledge-base and they lack adequate semantic interoperability, as similar names may be used with different meanings and *vice versa *even in indicator' sets developed and used in the same country. Furthermore, there is no international consensus regarding basic indicators for the evaluation and follow-up of mental health systems, and multiple sources of information are available at the international, national, regional and local levels, including health administration registers and large databases, health surveys, health statistics, commissioned reports, and key contacts or demographic censuses.

Although the available international instruments do provide a useful source of indicators (e.g. WHO-AIMS [5] or the Mental Health Country Profile [6]), these listings have not been developed as knowledge bases and their taxonomy and hierarchy has not been formalised in an explicit way. An indicator base may allow to select indicators from this base for specific uses in studies, projects and plan monitoring, as well as in specific services, programmes or target populations.

In 2008, the Clinical Management Working Group of the Spanish Society of Psychiatry (known by its Spanish acronym GClin-SEP) started the development of a preliminary taxonomy and a related knowledge-base of mental health indicators which would facilitate a future standard indicator set which could be used for inter-regional comparison in Spain, related to the National Health System Mental Health Strategy [7], taking into account the challenges and problems previously described [8,9].

As a first step a preliminary taxonomy and a related knowledge-base for mental health indicators in Spain was planned. A taxonomy may be defined as a particular classification arranged in a hierarchical structure providing supra and subtype relationships. Within the health care technology field a health knowledge-base is 'a system of storage, classification and presentation of relevant health information which includes databases, glossaries, articles, presentations and other documents regarding a specific health area or subject' [10]. This should assist the development of a list of basic indicators which would, in turn, facilitate informed evidence in mental health planning.

## Methods

This project is aimed to developing a conceptual map and a knowledge-base of mental health indicators suitable for mental-health planning which permits inter-regional comparisons, follow-up and evaluation of the health systems that currently exist in Spain. For this a mixed qualitative method was followed using frame analysis and nominal groups.

Frame analysis is a broadly defined method of enumerating and defining ideas and themes within a larger topic that is particularly useful for formalising concepts [11]. Of the four components of frame analysis, we focused on "frame bridging," which manifested as collaborating with experts who are interested in topic but do not commonly interact due to different training backgrounds or other reasons, and on "frame amplification," or the clarifying and elaborating of a framework from which to think about the issue of discussion [12]. Frame analysis has previously been applied to a wide range of social and health-related topics, such as consensus-building in online special-interest advocacy groups and understanding of the culture of nurse mangers [13]. Two members of the core group with a background in mental health system research (LS-C), and mental health geography and data management (JAS), searched the relevant literature in PubMed and Google Scholar using the key words: 1) "Mental Health", 2) "Care", "System", "Policy", "Planning" and 3) "Indicator(s)"; as well as a review of other technical documents available such as lists of general indicators of health relevant to mental health, and mental health lists from international, European and national organisations. Also considered were various plans and health reports from the Autonomous Communities or regions in Spain and lists developed by scientific associations. As the aim of this project was to develop a taxonomy usable in Spain within the European context, indicator lists from the US were not included in the analysis. The two researchers arranged this content according to key topics and prepared a framing document and a list of key areas and questions to be debated by the nominal groups.

The nominal group technique helps to deal with ill-structured domains while it allows a more structured approach than focus groups, as well as the use of prior information and knowledge. Once ideas and related questions are listed, its relevance to the central problem can be discussed following a question made by the facilitator, ideas can be re-formulated and clustered into coherent groups. All members are encouraged to participate in the discussion following a sequential order and every round is followed by a final debate [14,15]. In the health sector nominal groups have been previously used to develop the preliminary taxonomy of health related habits and lifestyle [16] and its integration into primary care [17].

An iterative process was followed to develop the preliminary taxonomy and the related knowledge-base. In all 14 experts in mental health service research and indicator analysis with very different background participated in two nominal groups: a core working group and an external group. The core working group was comprised of seven members: four psychiatrists with experience in the evaluation and management of services, one expert in data-analysis (Knowledge Discovery from Data -KDD) [3], a health geographer, and an expert in health and social management in the field of mental health. The core group hold three face-to-face meetings in 2009 and 2010, combined with three conference calls and periodic contact by e-mail.

Additionally, a panel of experts from the Scientific Association PSICOST provided external support to this core group. This external panel had seven members, a coordinator (LS) and a moderator (JAS). The panel also followed a nominal group methodology and it was comprised of two psychiatrists (LS and JCG), one psychologist (CR) with experience in services evaluation, a public health expert in epidemiology (JA), an expert in health-indicator data analysis (CG), and a public administration manager with experience in mental health and disability (FA).

For the development of this taxonomy the model and terminology used at the International Classification of Functioning (ICF) [18] was adopted for defining health constructs, domains and dimensions. For the definition of entities, their hierarchy and type, we used a basic formal terminology: <it is a>, <it is comprised of>, <it is part of>. A conceptual map was drafted using a tree structure for coding and organising the indicators. This approach had been used previously for the description of resource indicators and the use of mental health services in Spain and in other European countries [19]. This diagram allows the organisation of indicators into classes (domains), subclasses (subdomains) and additional types. This structure allows the addition of new indicators or the subdivision of previously defined indicators where necessary, without altering the hierarchical structure of the taxonomic system.

Subsequently, the two reviewers developed a list of relevant databases, a wide-ranging list of mental health indicators, and a glossary. With respect to the database, and bearing in mind all the information available, the following question was formulated for the nominal expert panel: "Is this a relevant indicator for the evaluation of the mental health system in the various Autonomous Communities?". 'Relevant' was defined here as 'closely connected with the subject and valuable and useful to mental health planners and stakeholders' based on the definition provided by the Oxford Advanced Learner's Dictionary http://www.oxfordadvancedlearnersdictionary.com. The responses were organised into a 4-level Likert scale according to their relevance (none, doubtful, moderate and high).

The results were reviewed by the members of the working group and the information gathered was used to develop a definitive list which was added to the preliminary knowledge base and which can be seen at the Spanish Society of Psychiatry website SEP [20]. The external nominal panel provided an evaluation of the relevance of the various indicators which was reviewed by the core working group.

## Results

### Document basis

Fourteen bases of relevant indicators were identified for the evaluation of mental health systems in Spain. These are shown in Table 1.

**Table 1:** Documents included in the Spanish mental health indicator knowledge base developed by GClin-SEP.

Ambit (levels)	Field	Year	link
**International**			

Assesment Instrument for Mental Health Sytems - WHO-AIMS	Mental health	2005	http://www.who.int/mental_health/evidence/WHO-AIMS/en/

Multi-country Survey Study on Health and Health's Systems Responsiveness - WHO Responsiveness	General health	2005	http://www.who.int/responsiveness/en/

Health Care Quality Indicator - OCDE	Mental health	2004	http://www.oecd.org/health/hcqi

**European**			

Policies and practices for mental health in Europe - WHO Europe	Mental health	2008	http://www.euro.who.int/en/what-we-do/health-topics/diseases-and-conditions/mental-health/publications/2008/policies-and-practices-for-mental-health-in-europe

European Community Health Indicators Monitoring - ECHIM	General health	2008	http://www.echim.org/

European Observatory on Health Systems and Policies	General health	2008	http://www.euro.who.int/observatory

Mental Health Information and Determinants for the European Level- MINDFUL (Stakes)	Mental health	2006	http://info.stakes.fi/mindful/EN/frontpage.htm

Mental Health Economics European Network - MHEEN	Mental health	2008	http://www.mhe-sme.org/mheen.html

**National**			

Key Indicators in the National Health System - INCLASNS-DB	General health	2007	http://www.sensefums.com/organizacion/sns/planCalidadSNS/t01.htm

The National Health System Mental- Health Strategy	Mental health	2007	http://www.msps.es/ciudadanos/saludMental/home.htm

Atlas of Variations in Medical Practice in the National Health System - Atlas VPM	General health	2008	http://www.atlasvpm.org/avpm/

Mental Health Observatory AEN	Mental health	2005	http://www.observatorio-aen.es/

Scientific Association PSICOST	Mental health	2003	http://www.edesdeproject.eu

**Regional (Autonomous Communities)**			

Mental Health Plan of Catalonia	Mental health	2006	http://www.gencat.cat/salut/depsalut/html/ca/dir489/index.html

### Preliminary taxonomy

**Figure 1:**
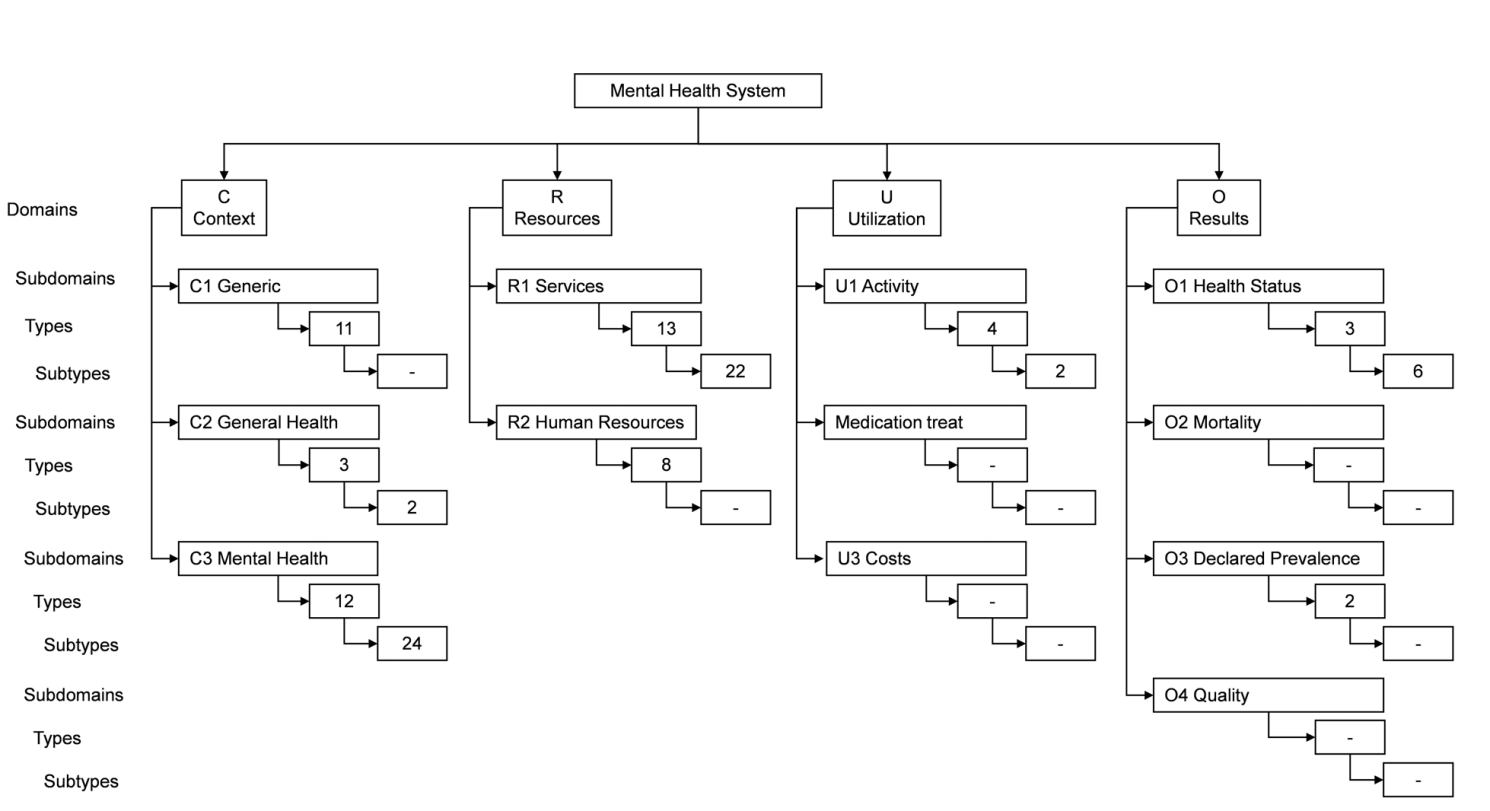
**Conceptual map of the hierarchical classification model of mental health indicators**.

For the hierarchical organisation of the classes, a tree structure has been used with four main branches corresponding to 'Context', 'Resources', 'Utilization' and 'Results'. Given the possibility of the taxonomy being used internationally, the decision was made to label them using their English initials (C: Context, R: Resources, U: Utilization, O: Outputs). The conceptual map is represented in Figure 1.

Table 2 details the indicators organised hierarchically into Domains, Subdomains, Types and Subtypes, along with their corresponding code. The *Mental Health System Context *domain contains three subdomains: *Generic Context *is, in turn, comprised of eleven types, *General Health Context *of three types and *Mental Health Context *of twelve types. The *Mental Health Resources *domain contains two subdomains: *Mental Health Services *with thirteen types and *Human Resources (personnel/staff) *with eight types. The *Utilization *domain has three subdomains: *Activity *with four types, *Medication treat*, and *Costs*. Finally, the *Results *domain is comprised of four subdomains: *Health Status *(containing, in turn, three types), *Mortality*, *Prevalence *(with two types), and *Quality*.

**Table 2:** Classification, hierarchical organisation and evaluation of the relevance of mental health indicators.

Code CBI-SEP	Denomination	Number of indicators				
	Relevance(*)	***High***	***Moderate***	***Doubtful***	***No rel***.	**Total**
**C**	**CONTEXT DOMAIN**	**66**	**83**	**73**	**95**	**317**

**C1**	**GENERIC**	**13**	**16**	**19**	**2**	**50**

C1.1	Population structure	3	3			6

C1.2	Births			5		5

C1.3	Migration	1	1	3		5

C1.4	Civil status	1	2			3

C1.5	Education		1	4		5

C1.6	Childhood		1	1	1	3

C1.7	Households	3				3

C1.8	Regional distribution	2	1			3

C1.9	Population development		1	1		2

C1.10	Macroeconomy	2		5		7

C1.11	Employment	1	6		1	8

**C2**	**General Health**	**4**	**10**	**4**	**1**	**19**

C2.1	Financing	4	5	2		11

C2.1.1	General financing	2	3	2		7

C2.1.2	Medication expenditure	2	2			4

C2.2	Health status		4			4

C2.3	Mortality		1	2	1	4

**C3**	**Mental Health**	**49**	**57**	**50**	**92**	**248**

C3.1	Regulations	1	1	6	9	17

C3.1.1	Policy				4	4

C3.1.2	Plans	1		3		4

C3.1.3	Legislation		1	3	5	9

C3.2	Financing	8	23	2	7	40

C3.2.1	General financing	3	9	2	3	17

C3.2.2	Medication expenditure	5	14		4	23

C3.3	Management and organisation	8	2		1	11

C3.4	Territorial zoning	3	3	1		7

C3.5	Information systems	3	6	1		10

C3.6	Care Services listing	10	5	1	18	34

C3.6.1	Services	10	5	1	7	23

C3.6.2	Eligibility				6	6

C3.6.3	Medication listing				5	5

C3.7	Coordination	6	1			7

C3.8	Primary Healthcare	2	4		18	24

C3.9	Prevention and promotion	5			31	36

C3.9.1	General	3			1	4

C3.9.2	Education	1			9	10

C3.9.3	Employment	1			4	5

C3.9.4	Stigma and sensitisation				3	3

C3.9.5	Programmes by age groups				3	3

C3.9.6	Suicide prevention				4	4

C3.9.7	Prevention of depression				5	5

C3.9.8	Minorities				2	2

C3.10	Research	2	9	1		12

C3.10.1	Financing		3			3

C3.10.2	Organisation		4			4

C3.10.3	Results	2	2	1		5

C3.11	Training			17	5	22

C3.11.1	Mental health training				5	5

C3.11.2	Lifelong learning			7		7

C3.11.3	Lifelong learning in special groups			10		10

C3.12	Other	1	3	21	3	28

C3.12.1	Human Rights			10		10

C3.12.2	Participation/empowerment	1	3	11	3	18

C3.12.2.1	User & family organization	1	3	1		5

C3.12.2.2	Financing			4	3	7

C3.12.2.3	Coordination			6		6

**R**	**RESOURCES DOMAIN**	**101**	**23**	**59**	**4**	**187**

**R1**	**Services**	**71**	**6**	**56**	**3**	**136**

R1.1	Hospital and residential care	2	1		1	4

R1.1.1	Hospital and residential services	1				1

R1.1.2	Beds in hospital and residential services	1	1		1	3

R1.2	Hospital acute-unit care	6				6

R1.2.1	Hospital acute-unit services	3				3

R1.2.2	Beds in acute-unit hospital services	3				3

R1.3	Hospital care for non-acute patients	2		4		6

R1.3.1	Hospital services for non-acute patients	1		4		5

R1.3.2	Beds in hospital services for non-acute patients	1				1

R1.4	Residential care for acute patients	2				2

R1.4.1	Residential services for acute patients	1				1

R1.4.2	Beds in residential services for acute patients	1				1

R1.5	Residential care for non-acute patients	4		9		13

R1.5.1	Residential services for non-acute patients	2		9		11

R1.5.2	Beds in residential services for non- acute patients	2				2

R1.6	Day care	14	4	15		33

R1.6.1	Daycare services	7	2	15		24

R1.6.2	Places in daycare services	7	2			9

R1.7	Out-patient care	5	1	20		26

R1.8	Forensic psychiatric care	2			1	3

R1.8.1	Forensic psychiatric services	1				1

R1.8.2	Beds in forensic psychiatric services	1			1	2

R1.9	Infant-adolescent care	7		2	1	10

R1.9.1	Infant-adolescent services	4				4

R1.9.2	Beds and places in infant-adolescent services	3		2	1	6

R1.10	Psychogeriatric care	10				10

R1.10.1	Psychogeriatric services	6				6

R1.10.2	Beds and places in psychogeriatric services	4				4

R1.11	Drug-dependence care	12				12

R1.11.1	Drug-dependence services	6				6

R1.11.2	Beds and places in drug-dependence services	6				6

R1.12	Intellectual disabilities care	5				5

R1.12.1	Intellectual disabilities services	3				3

R1.12.2	Beds and places in intellectual disabilities services	2				2

R1.13	Information and accessibility to care			6		6

**R2**	**Human Resources**	**30**	**17**	**3**	**1**	**51**

R2.1	Total mental health professionals	10	1	1		12

R2.2	Psychiatrists	5	3	1	1	10

R2.3	Psychologists	5	2			7

R2.4	Postgraduate trainees (psychiatrists. & psychol)ogists)		2			2

R2.5	Nursing staff	5	2	1		8

R2.6	Social workers	5	2			7

R2.7	Nursing assistants		4			4

R2.8	Administrative staff		1			1

**U**	**UTILIZATION DOMAIN**	**17**	**31**	**8**	**11**	**67**

**U1**	**Activity**	**16**	**24**	**6**	**10**	**56**

U1.1	Visits/contacts	3	2	1		6

U1.2	Users	5	16	2	8	31

U1.2.1	Treated prevalence	2	16		2	20

U1.2.2	Patient groups	3		2	6	11

U1.3	Discharges	5	6	2		13

U1.4	Mean stay	3		1	2	6

**U2**	**Medication treat**		**7**		**1**	**8**

**U3**	**Costs**	**1**		**2**		**3**

**O**	**RESULTS DOMAIN**	**16**	**22**	**52**		**90**

**O1**	**Health status**	**6**	**22**	**5**		**33**

O1.1	Reported outcomes		19			19

O1.1.1	General quality-of-life		2			2

O1.1.2	Functional status		3			3

O1.1.3	Clinical status		6			6

O1.1.4	Other		8			8

O1.2	Adjusted Life Years Indexes		2	2		4

O1.3	Official Declaration of Disability	6	1	3		10

O1.3.1	Work-related	2		2		4

O1.3.2	Non Work-related	4	1	1		6

**O2**	**Mortality**	**5**				**5**

**O3**	**Prevalence**	**5**		**9**		**14**

O3.1	General population	5		6		11

O3.2	In the crime & justice system			3		3

**O4**	**Quality**			**38**		**38**

	**TOTAL**	**200**	**159**	**192**	**110**	**661**

* Closely connected with the subject and valuable and useful to mental health planners and stakeholders

A detailed description of the typology of the mental health system indicators can be seen in the database at the Spanish Psychiatric Society website [20].

### Knowledge base components

The knowledge base developed by the working group consists of the list of the relevant indicator bases with their links, as well as a database of indicators and a glossary appendix.

The mental health system base of indicators is composed of 661 indicators organised according to the proposed taxonomy. The definition of each indicator was developed using cards which containing the name, the unit of measurement and calculation, source, and availability at the geographical area. Evaluation of the relevance of the various indicators by the nominal panel, reviewed by the core working group, can be seen in Table 2. This evaluation has allowed identification, in accordance with their relevance for the mental health system in Spain, 200 high-relevance indicators, 159 of moderate relevance, 192 of doubtful relevance, and 110 of no relevance to the aim of this list.

## Discussion

The present work is framed in the context of informed evidence for health policy and planning [21]. The concept of informed evidence is replacing that of evidence-based care and highlights the need for quality registers and the greatest possible number of information sources available for decision-making in health policy, including local provision and organisation at different levels (micro, meso and macro) [22].

To our knowledge this is the first preliminary taxonomy of indicators of the mental health system and its related knowledge-base. Other preliminary taxonomies have been recently developed to formally organise other areas of knowledge such as health indicators [23], patient safety and medical errors [24], or health related habits [16]. This preliminary taxonomy does not pretend to develop a completely different conceptual map to what is currently used in the field, but to formally organise the available information and provide a hierarchical order using common terminology as much as possible. The definitions selected were also those more commonly accepted. The extent of the area of health indicators is such that it hinders a complete review of the material; especially for a restricted group with a limited budget. This knowledge base has an incomplete character and several limitations.

First, this knowledge-base is country-specific and its generalisability and transferability outside Spain is limited. In any case it is important to note that country-level information is very relevant for international health system research [22]. Furthermore, the heterogeneity of the Spanish mental health system makes it a unique case for studying different care models under quasi- or universal health care coverage. The existence of 17 different publicly funded mental health systems, with their own policy and practices may provide useful insight for many countries. They range from a practically do-nothing approach until very recently in some regions, to the transformation of the old psychiatric hospitals, complete separation of funding and provision, with market competition and high participation of the private sector working under agreements set by the public health system (e.g. Catalonia). They may have one single public system (comprising both funding and provision) without closure of psychiatric hospitals (e.g. Basque Country) or a public system with full deinstitutionalisation and closure of psychiatric hospitals (e.g. Andalusia) [25]. In addition the conceptual map included in this preliminary taxonomy has been designed to facilitate the incorporation of new domains and sub-domains as the system is expanded, so it may be refined when information from other sources is incorporated (e.g. user-oriented mental health report cards), or when it is used in other countries in Europe.

Second, this knowledge base is expert-oriented and it has excluded international indicator lists not developed or used in Spain, such as the Mental Health Country Profile [6]; the NF-10 and its related instruments in the US [26] or the 'State Report Cards' by the National Alliance on Mental Illness (NAMI) [27]. As said, this knowledge-base should be complemented by user-oriented indicator lists based on concerns reported by consumers which are not currently available in Spain.

Third, there are great differences regarding the degree to which this information can be accessed. The majority of indicators are available on a national and regional scale but these are limited in small health areas. The limitations of scale, periodicity and sources mean that some indicators cannot be selected despite their potential relevance. The sources of information for the calculation of the indicators are highly heterogeneous with the institutes of statistics, and information from health administrations and social welfare being the principle sources of data. Furthermore, the reference year of these sources varies across the 17 regions or Autonomous Communities in Spain, and even within the same Autonomous Community. This is related to the fact that, after the devolution process started in 1986, the Spanish Health System actually comprises 17 different health systems with wide variation in mental health care organisation and policy [25].

Fourth, the extended list included important indicators that have not been incorporated to the 200 indicator list due to usability problems in the Spanish case. These comprise patient reported outcomes, stigma and sensitation, suicide prevention, prevention of depression, training and human rights. To date human rights have been specifically assessed in a single region (Asturias), and results have not been published yet. In any case a list of 200 indicators is too large to be practical for decision making, even though other main lists and instruments contain a similar number of indicators (e.g. WHO-AIMS [5]). The OECD list comprises 12 indicators which are included in the expanded GClin-SEP list. Unfortunately just one is currently collected in Spain [28]. GClin-SEP is conducting a Delphi panel on the relevance and usability of these indicators to produce a brief list of 50 indicators usable for comparing mental health systems across the 17 regions, and for the standard monitoring of the Spanish National Mental Health Strategy. This Delphi study will provide data on the feasibility and face validity of the indicators registered in this listing.

In addition, there is scant information on the psychometric properties of the indicators in the care system [2]. The development of a preliminary taxonomy is complementary to the psychometric analysis of the indicator set. Health system indicators are very basic health technology tools, and hence, their feasibility, consistency, validity, reliability, redundancy, sensitivity to change, level of generalisability, and impact analysis should be evaluated following standard procedures [29]. The existing gap between the literature on the psychometric properties of indicators and its broad use in health service and health system research may be partly related to a lack of awareness by researchers, planners and funding agencies of the relevance of this topic and the need for additional funds in this field.

## Conclusion

This preliminary taxonomy and its related knowledge-base should serve those embarking on a study of the Spanish Mental Health System, and it may be also valuable to researchers looking for selected indicator lists in specific areas within mental health system research in Spain. It may be also relevant as a contextual case to those analysing indicator lists in other countries, particularly in Europe. On the other hand the preliminary taxonomy and its related conceptual map and hierarchy would require comparison with other related international initiatives and further analysis following a formal ontology approach [23]. These results should be challenged in other European countries to improve the indicators on Mental Health Systems in this world region.

## Competing interests

The authors declare that they have no competing interests.

## Authors' contributions

AB coordinated the project. LSC and JAS prepared the framing document, managed the nominal groups and wrote the draft. MM, MG, KG and MR participated in the core group and reviewed the draft and related documents. All authors read and approved the final manuscript.
